# Plastic Devolatilisation Kinetics During Isothermal High-Temperature Pyrolysis: Focus on Solid Products (Part I)

**DOI:** 10.3390/polym17040525

**Published:** 2025-02-18

**Authors:** Ieva Kiminaitė, Sebastian Wilhelm, Lukas Martetschläger, Clara Leonie Brigitte Eckert, Marcos Berenguer Casco, Nerijus Striūgas, Sebastian Fendt

**Affiliations:** 1 Laboratory of Combustion Processes, Lithuanian Energy Institute, 44403 Kaunas, Lithuania; nerijus.striugas@lei.lt; 2 Chair of Energy Systems, Department of Mechanical Engineering, Technical University of Munich, 80333 Garching, Germany; sebastian.g.wilhelm@tum.de (S.W.);; 3 TUM School of Engineering and Design, Technical University of Munich, 80333 Garching, Germany; 4 School of Industrial Engineering, The Polytechnic University of Valencia, 46022 València, Spain; m.bercas@etsii.upv.es

**Keywords:** plastic packaging waste, flash pyrolysis, devolatilisation, carbon black, single first-order reaction model, kinetic modelling, activation energy, compensation effect

## Abstract

Incineration remains Europe’s main practice for plastic packaging waste treatment, primarily due to the limitations of mechanical recycling technology. Consequently, research and development of more sustainable and flexible approaches are of high importance. Thermochemical conversion of polypropylene, polystyrene, and municipal plastic packaging mix via high-temperature flash pyrolysis (1000 °C/s) is studied in this research, focusing on the kinetics and yields of the devolatilisation stage. The primary stage results in the formation of volatile organic compounds considered intermediate products for carbon black production. The experiments were conducted in a pressurised wire mesh reactor, investigating the influence of temperature (600–1200 °C), residence time (0.5–10 s), and pressure (1–25 bar). The positive effect of temperature on the volatile yield was observed up to 2–5 s. The devolatilisation stage was completed within a maximum of 5 s at temperatures ranging from 800 to 1200 °C. The pressure was determined to be a kinetically limiting factor of the process to up to 800 °C, and the effect was not present at ≥1000 °C. Raman spectroscopy measurements revealed that pyrolytic carbon deposited on the post-experimental meshes is structurally similar to the industrially produced carbon black. The kinetic data and developed model can be further applied in the upscale reactor design.

## 1. Introduction

**Figure 1:**
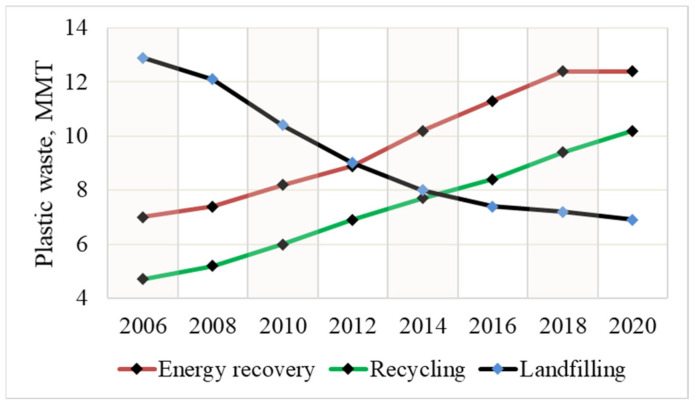
Plastic waste treatment methods in EU27+3 from 2006 to 2020 [3].

Sustainable handling of plastic waste (PW) remains a critical challenge in the 21st century. Extensive consumption of single-use plastic products, non-biodegradability, and economic and technical barriers in recycling processes are the main factors contributing to PW accumulation and environmental pollution [1]. According to Our World in Data, approximately 450 million tons of plastic waste is generated annually worldwide [2], with around 50 million tons in Europe. Figure 1 summarises the trends of PW treatment methods in EU27+3 in 2010–2020. Yet, heat energy recovery through incineration serves as the main strategy [3], which is unappealing due to the excessive generation of greenhouse gases and toxic substances [4]. On the other hand, a high share of PW is still directed to landfill disposal, which severely contaminates the natural biosphere and is considered a waste of resources [5]. Notably, the rate of PW recycling into secondary materials has shown a positive trend in recent decades. Nevertheless, heat energy recovery with the major contribution to CO_2_ emissions remains the main practice of PW treatment in Europe, highlighting the necessity of PW management improvement.

Among global plastics markets, the packaging sector holds the largest share, accounting for 44% [3], which results in an immense waste stream that is challenging to recycle mechanically. Mechanical or secondary recycling is the most common approach for recycling plastic into secondary materials [6], covering a mere 10% of the PW stream in Europe [3]. Secondary recycling relies on homogenous plastic streams and thus requires precise sorting and decontamination of municipal PW. Concurrently, polymers are subjected to thermo-mechanical and thermo-oxidative degradation during this recycling process, leading, in particular, to changes in their chemical structure and subsequent deterioration of mechanical properties [7]. Consequently, this approach is limited in sufficient recycling complex PW. The recyclability rate also varies significantly depending on the plastic type used. For example, polystyrene (PS) and polypropylene (PP) are reported to have one of the lowest recyclability rates due to their low density [8] and high post-consumer contamination, together with chemical structure deterioration during treatment [9]. The solution lies in minimising waste and developing more flexible recycling or upcycling technologies to obtain value-added products to reduce PW and contribute to the development of the circular economy.

Along with the need to further develop plastic recycling technologies, the low-carbon footprint techniques of upcycling complex PW into value-added products (e.g., chemicals, solvents, functional carbon materials, and others) will be an additional pathway. Advantageously, thermochemical conversion processes, such as pyrolysis and gasification, are neutral to post-consumer contamination of PW in the production of a wide range of hydrocarbons; however, contaminants (e.g., lead, chlorine, etc.) may still be problematic in terms of corrosion and fouling of the reactor system; this needs to be addressed [10]. This study presents a novel approach to PW valorisation by recovering carbon black (CB), focusing on the first stage of the process—devolatilisation. Carbon black, also called furnace black, is conventionally produced in a turbulent flow horizontal furnace at 1200–1900 °C via partial combustion, with a residence time of 10 s to 10 ms according to the conversion temperature. Natural gas serves as a fuel, and petrochemical oil is used as a feedstock [11]. The furnace process constitutes more than 95% of total CB produced worldwide, with a predominant use as a reinforcing agent in rubber tyres and as a pigment for inks, coatings, and other applications [12]. The negative aspect of this process is the particularly high carbon footprint—from 1.90 to 5.25 kg of carbon dioxide per 1 kg of CB [13]. Many authors have proposed high-temperature pyrolysis of hydrocarbons as a sufficient decarbonisation approach by producing hydrogen gas and carbon black, commonly using methane as a feedstock [14]. Most plastic pyrolysis studies use catalysts targeting filamentous carbon and hydrogen gas generation via chemical vapour deposition. The primary benefit of transition metal catalysts is the significantly lowered process temperatures, i.e., from around 1300 °C to 500–950 °C, as reported by S. Ahmed et al. [15]. However, amorphous carbon black is typically produced via non-catalytic thermal or plasma technologies. In the 1990s, the Kvaerner Company attempted to apply CO_2_-neutral plasma technology via thermal decomposition of hydrocarbons to H_2_ gas and CB by hydrogen plasma [16]. However, further development of the process was stopped due to technical issues in the quality control of the CB [17]. On the other hand, plasma pyrolysis of plastics is an extensively investigated topic. For instance, K. P. Bhatt et al. [18] reported high efficiency of thermal hydrogen plasma decomposition of PET, LDPE, HDPE, and PP at 700–1000 °C, where hydrogen-rich gas was the main product, with solid carbon formed to a lower extent, reaching 5–17% yield depending on the feedstock and temperature. Here, we raise the hypothesis that to improve the decarbonisation targeting CB formation, a two-stage process should be adopted, first decomposing the plastic material (primary cracking) and then subjecting the intermediate hydrocarbons to thermal plasma. This study chose a single-stage approach of high-temperature pyrolysis (thermal path), where PW is considered a hydrocarbon feedstock.

During plastic pyrolysis, thermoplastic polymers initially melt when heated to the material-specific melting temperature. Upon further heating, the polymers decompose, rapidly releasing volatile organic compounds (VOCs). It is generally accepted that polyolefins decompose through a free-radical chain reaction mechanism. This process begins with the random cleavage of C-C bonds, leading to the formation of free radicals. At elevated temperatures, the free radicals are highly reactive with one another and with neutral molecules, triggering various secondary and tertiary reactions [19]. The process conditions, i.e., temperature, heating rate, atmosphere, residence time, and pressure, essentially determine the mechanism of subsequent chemical reactions and product distribution. R. K. Singh et al. [20] reported that post-consumer municipal plastic waste is converted to diesel-like oil through non-isothermal pyrolysis at slow heating for 10–20 °C/min. In contrast, iso-thermal conditions at 500 °C with a heating rate of 20 000 °C/s favoured the formation of gaseous products. Previous studies on flash pyrolysis of PP and PS feedstocks highlighted the differences in evolved VOC composition from feedstocks of differing chemical structures. For example, M. M. Win et al. [21] showed that PP at 900 °C flash pyrolysis decomposes into light hydrocarbons (CH_4_; C_2_H_n_; C_3_H_n_), accounting for ~50%, and tars, accounting for less than 10%, while PS plastic, made of aromatic hydrocarbon styrene via pyrolysis at 825 °C in the free-fall reactor, decomposes by releasing styrene monomeric units, with a maximum yield of 34%. Higher temperatures increase the yield of light hydrocarbons, depending on the vapour residence time [22]. Residence time or reaction time is a parameter that is precisely controlled in CB production processes due to its major influence on CB particle size, graphitisation, and mechanical properties [11]. The highest coking rate of toluene feedstock (33.4 g/m^2^·h) was established at 950 °C in 1.2 s residence time, achieving 40% conversion as reported by F. Albright and J. C. Marek [23].

It is known that aromatic hydrocarbons play an important role in soot and CB formation. The temperature elevation was reported to positively affect the formation of aromatic hydrocarbons within a reaction time of 1 min during plastic pyrolysis due to the Diels–Alder reaction [24,25]. Subsequent polycondensation results in the formation of polycyclic aromatic hydrocarbons (PAHs) and CB when residence time is increased, influencing the aggregate size as well [26]. The formation of higher hydrocarbons, including aromatic species, can also be promoted by elevated pressure of up to 19.2 MPa, as presented by P. T. Williams & E. Slaney [27]. To address the effect of pressure on the volatile yield and coke formation, the influence of up to 25 MPa of pressure was included and defined in this investigation.

The volatile yield and kinetics of the primary cracking of plastics during flash pyrolysis are important parameters in the pyrolysis reactor design. The global kinetic models simplify the reaction mechanism to one or a couple of reactions capturing the most important features of the process [28]. Kinetic parameters of the process of undefined reaction order are determined using model-free approaches for experimental data under isothermal or non-isothermal conditions [29]. The vast majority of previous pyrolysis kinetic studies on different types of polymers were aimed at evaluating kinetic parameters on non-isothermal conditions via thermogravimetric analysis by isoconversional methods [30,31,32,33,34,35,36,37]. These are based on the calculation of Arrhenius parameters—activation energy E_α_, frequency factor or pre-exponential factor A—for a specific conversion value (α) by obtaining gravimetric data at different heating rates (β), commonly in the range of 5–40 °C/min. Using this method, estimated average values of activation energy for plastic conversion fall within the broad range of 87–290 kJ/mol [30,33,34,35,37,38,39,40,41], explained by the kinetic compensation effect. Despite the convenience of using thermal analysis to study pyrolysis kinetics, the main drawbacks noted by other authors are slow heating rates and mass transfer limitations [42,43]. The devolatilisation model in this study aligns with the entrained flow pyrolysis conditions, where plastic particles, hypothetically fed through a cooled feeding tube (to prevent melting), are instantaneously exposed to the reaction temperature. Therefore, experimental conditions in this study correspond to the industrial pyrolysis reactors targeting CB formation (flash thermal decomposition at 1200–1900 °C) [11], including lower temperature points according to previous studies on the conversion of hydrocarbon feedstocks [21,23]. Apart from the effect of temperature on reaction kinetics, the influence of heat transfer mode and particle size must also be addressed. A. Niksiar et al. [44], in their investigation of PET plastic pyrolysis in a spouted bed reactor and TGA, showed that activation energy decreases from 276.8 to 264.3 kJ/mol as particle size increases from 0.1 to 3.0 mm. An equivalent effect was confirmed by P. Paik and K. Kar [45], who found that the activation energy and lifetime of nanosized PP particles were moderately higher than those of microsized PP particles. Additionally, the type of heat energy transfer, which controls the heating rate of the sample and kinetic control along with product distribution, also plays an important role [46]. It is accepted that heat radiation results in the highest conversion rate [47].

This research aims to define the impact of temperature, pressure, and holding time on the rate and kinetic limitations of the first stage of the process—devolatilisation is a correlative to volatile yield during isothermal pyrolysis of PP, PS, and PW mix plastics. VOCs evolved during plastic pyrolysis are considered intermediate products involved in secondary and tertiary reactions in carbon black formation and were not considered in this study. Additionally, the structural properties of pyrolytic carbon deposited during flash pyrolysis were investigated.

## 2. Materials and Methods

### 2.1. Preparation of Plastic Samples

Polypropylene (PP), polystyrene (PS), and the municipal plastic packaging waste mix (PW mix) collected in Garching, Germany, were investigated in this study. Isotactic PP and PS plastic pellets were purchased from Sigma-Aldrich^®^ (Darmstadt, Germany) and milled cryogenically. PW mix before cryo-milling was washed with distilled water and dried at 80 °C for 12 h. The milled plastics were passed through a 1 mm mesh sieve to ensure an even distribution of particle size. The composition of the samples was characterised via proximate (ASTM D3172) [48] and ultimate (ASTM D3176) [49] standard analysis methods. The results and material information are presented in Table 1.

### 2.2. Thermal Analysis

Thermogravimetric analysis (TGA) was carried out in this study to evaluate the thermal decomposition characteristics of plastic feedstocks during the pyrolytic conversion. NETZSCH STA 449 F3 Jupiter analyser (equipment sourced from NETZSCH, Selb, Germany) was employed for the analysis. Samples were weighed in aluminium oxide crucibles; the average weight was 5 ± 1 mg. The temperature program was run from 40 °C to 800 °C at 10 °C/min under a constant N_2_ gas flow of 60 mL/min. Samples were maintained at the maximum temperature for 10 min. Weight change curves were corrected by automatically subtracting the blank curves—TGA curves obtained at the defined conditions with an empty crucible. The first derivate of the measured TG curve–differential thermogravimetric curve (DTG) was calculated for the rate of mass change estimation.

### 2.3. Flash Pyrolysis Experiments in Wire Mesh Reactor

**Figure 2:**
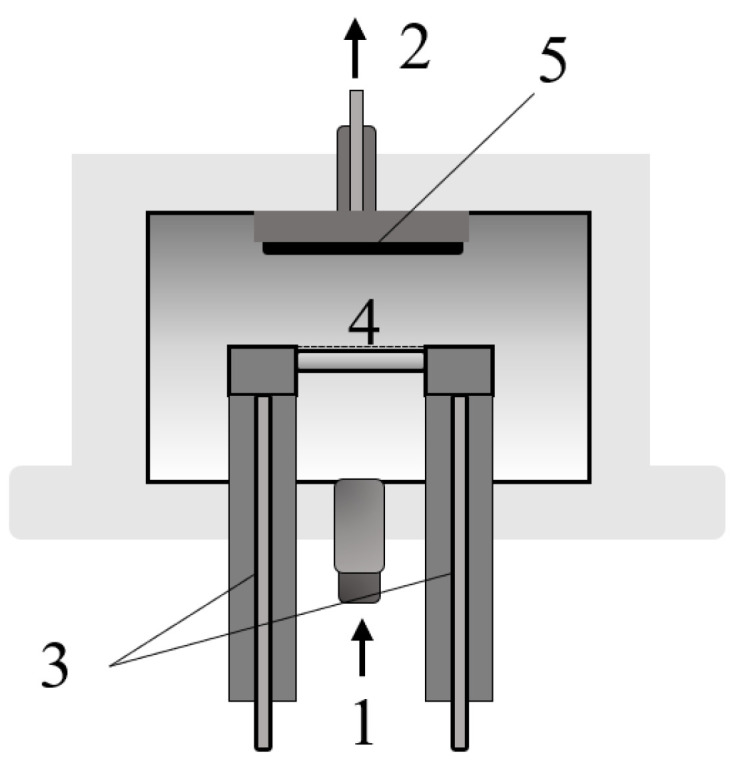
Scheme of the WMR: 1—gas inlet; 2—gas outlet; 3—copper electrodes; 4—wire mesh with the sample; 5—metal filter to trap tars.

Devolatilisation of plastic samples via isothermal pyrolysis was performed in the pressurised wire mesh reactor (WMR) at the Technical University of Munich, Garching, Germany. The reactor consists of two electrodes and a wire mesh clamped in between that acts as a resistance heater (number 4 in Figure 2) for Joule heating. A stainless steel wire mesh (AISI-314) with a 42 μm grid and a maximum operating temperature of 1200 °C was used. The mesh was welded to obtain a small 20 mm × 20 mm pocket for 30 ± 5 mg sample weight. Two type K thermocouple wires, nickel (negative) and nickel–chromium (positive), were welded onto the outer surface of the mesh to measure and regulate temperature. Resistance heating of the mesh was controlled via LabVIEW software (Version 8.6), adjusting the voltage supply. A schematic diagram of the reactor is given in Figure 2. The precise description of the experimental setup and operation is explained by A. Tremel [50].

**Figure 3:**
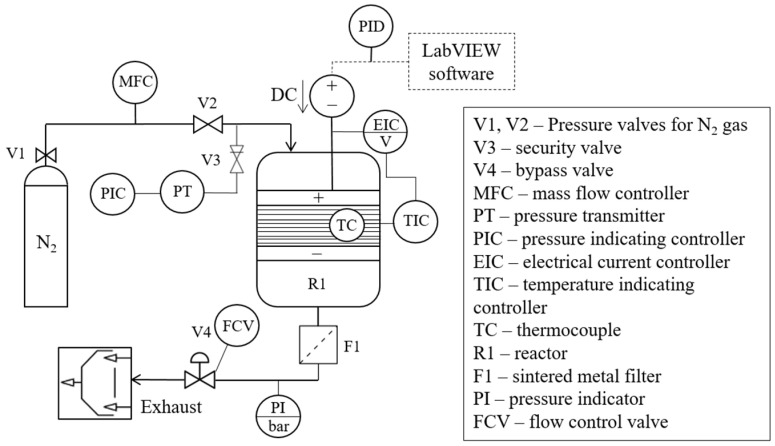
P&I diagram of the pyrolysis process in WMR experimental setup.

In addition to the setup shown in Figure 2, the experimental configuration (Figure 3) includes a gas supply, a control unit, and a power supply (SM 15–200 D, Delta Elektronika, 0–200 A, 0–15 V). The process parameters are controlled using the NI cRIO-9072 real-time controller and a control program in LabVIEW from National Instruments that features an integrated PID controller.

The flash pyrolysis experiments were carried out by heating the mesh with the sample at a 1000 °C/s heating rate. The influence of pressure on the devolatilisation was evaluated at a temperature ranging from 600 to 1000 °C, applying between 1 and 25 bar of pressure. N_2_ gas flow of 3.2 L/min was used for experiments in atmospheric pressure. To determine the influence of temperature and holding time on reaction rate and volatile yield, samples were held at 800, 1000, and 1200 °C for 0.5, 2, 5, and 10 s under atmospheric pressure. The yield of volatiles was determined by weighing the mesh with the sample before and after the experiment.

### 2.4. Modelling of Devolatilisation Kinetics and Volatile Yield

A first-order reaction model is commonly used for kinetic modelling of devolatilisation during pyrolysis. The model states that the reaction rate is directly proportional to the concentration of reactants—the yield of volatiles increases in a linear trend during the process until a plateau stage; thus, the dependence of the conversion rate and the release of VOCs on temperature and holding time can be defined with high accuracy and simplicity. A single first-order reaction model (SFOR) was chosen in this study as the most reliable model for PW pyrolysis; this was confirmed by previous research [51]. A simplified devolatilisation reaction of investigated plastics during pyrolysis, confirmed by thermal analysis (Section 3.1), is shown in Equation (1):

(1) $$Plastic\overset{k}{\to }Volatile\;organic\;compounds$$

The conversion rate (%/s) is then expressed as a function of reaction temperature influence described by the multiplication of temperature-dependent reaction rate constant *k* (Arrhenius equation) with the reaction model [29], Equation (2):

(2) $$\frac{d\alpha }{dt}=k\cdot f\left(\alpha \right)=A\cdot e^{-\frac{Ea}{RT}}\cdot (\alpha^{*}-\alpha_{t})\;$$

Here, *A* is the pre-exponential factor (1/s), *E_a_* is the activation energy (J/mol), *R* is the universal gas constant (8.315 J/mol·K), *T* is the pyrolysis temperature (K), α^*^ is the maximum volatile matter yield (specific to a feedstock material), and *α_t_* is the volatile yield after *t* duration of the experiment. The kinetic parameters *A* and *E_a_* are determined by the least square fitting with experimentally derived data.

The linear temperature dependency is expressed in Equation (3):

(3) $$\ln\;k=\ln A\cdot \left(-\frac{Ea}{RT}\right)$$

Equation (4) describes the influence of pressure on the pyrolysis volatile yield, where indirect proportionality between *dα/dp* and *p* is assumed [50]:

(4) $$\alpha \left(p\right)=\alpha_{p_{set}}-\frac{ln(p/p_{set})}{\rho }$$

Here, *α* (*p*) is the experimentally measured volatile yield at the studied pressure point, *α_set_* is the volatile yield at a set pressure, *p_set_* is the setpoint (minimum value of the pressure applied), *p* is the pressure value for the influence investigation, and *ρ* is an adjustable parameter, revealing the pressure influence on volatile yield. The adjustable parameter *ρ* is calculated by a least square fitting procedure using *p_set_* = 1 bar and the volatile yield experimental data points. The approach considers the reference pressure (*p_set_*) as the baseline where the yield is unchanged, with the pressure increase affecting the yield negatively in the logarithmic function. A low value of *ρ* represents a strong impact and vice versa. An established approach from reference research [50] was employed to determine the influence. To maintain scientific transparency and consistency, it is defined that high *ρ* values ≥ 100 indicate a low impact of pressure on devolatilisation, while low *ρ* values ≤ 1 indicate a high impact of pressure on devolatilisation. This classification aligns with prior studies that used ρ to estimate the sensitivity of volatile yield to pressure variations [50,52].

The parameters used for pyrolysis model development are presented in Table 2. The primary values of Arrhenius parameters were chosen following previous research on isothermal pyrolysis of plastics [53] and pressure influence, considering it to be low, taken from the reference by A. Tremel & H. Spliethoff [52].

### 2.5. Raman Spectroscopy Measurements of Pyrolytic Carbon Deposits

Structural characteristics of carbon deposits were investigated using Raman microscopy directly at the surface of post-experimental meshes due to the low residual weight of samples. The Raman spectra were recorded in the Raman shift range of 800−1800 cm^–1^ by Renishaw inVia™ Raman microscope (equipment sourced from Renishaw, Gloucestershire, UK) using an objective lens of 5× magnification. An objective lens of lower magnification was employed due to mesh deformation influenced by high temperature during the experiment. A 785 nm laser was used, applying 5% laser power and measuring 3 repetitions of the scan. The Raman measurements were repeated on three different spots of the meshes to obtain the average intensity values. The fluorescent background was not removed to avoid errors in the background removal process.

To evaluate the influence of pressure and temperature on the structural properties of carbon, meshes with pure plastics maintained at 1 bar and 25 bar for 10 s in the temperature range of 800–1200 °C were measured. Samples treated at 600 °C were excluded from the Raman measurements as the plastic conversion was incomplete in the maximal investigated holding time (10 s), as discussed in Section 3.2.

The reference material used for comparison was furnace black (produced by a conventional furnace process) purchased from Kremer Pigmente GmbH & Co. KG (Aichstetten, Germany).

## 3. Results and Discussion

### 3.1. Feedstock Thermal Analysis

**Figure 4:**
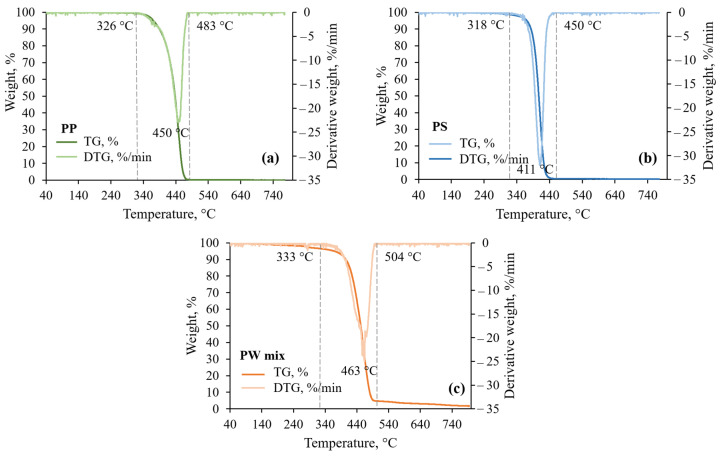
TGA and DTG curves of (**a**) polypropylene, (**b**) polystyrene, and (**c**) plastic waste mix at 10 °C/min heating rate, 60 mL/min N_2_ flow, and atmospheric pressure.

The thermograms in Figure 4 illustrate the decomposition patterns of analysed plastic samples during conventional, slow pyrolysis. Figure 4 marks the decomposition onset, the differential thermogravimetric curve (DTG) peak, and the decomposition offset temperatures of studied plastics. The thermal stability of the investigated plastics, determined by the onset temperature according to ASTM E2550 [54], decreases in the order PW mix > PP > PS.

The PP and PS polymers decompose in a single weight-loss step. The PW mix sample also decomposes in a major single stage, although the shoulder peaks indicate the thermal decomposition of several types of polymers present in the mixture.

Slow pyrolysis of investigated plastics resulted in a rapid release of volatile compounds without the carbonisation of PP and PS, while in the PW mix case, <5% of char formed, followed by degradation in the temperature range 500–760 °C. Generally, slow pyrolysis produces higher char yields from cross-linked polymers [55], e.g., lignin in biomass. Covalent bonding between polymeric chains increases the thermal stability of a polymer; consequently, such substances have high fixed carbon contents, and deposition of char is observed [56]. Thus, slow pyrolysis to convert PP, PS, and PW mix to char is not valid, and other approaches should be employed to recover carbon as a solid product. Overall, the thermal analysis results confirm that plastic pyrolysis occurs in a major single decomposition stage (feasibility of SFOR reaction model) with a volatile yield close to 100%.

### 3.2. Influence of Temperature and Holding Time on Volatile Yield During Flash Pyrolysis

**Figure 5:**
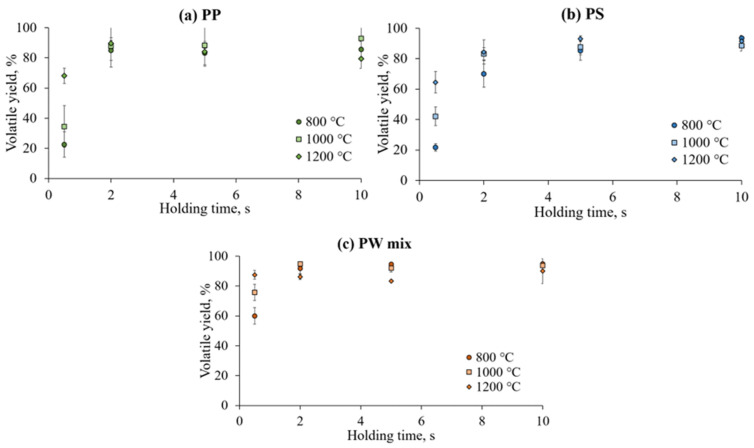
Volatile yields on wire mesh reactor, from 30 mg of (**a**) polypropylene, (**b**) polystyrene, and (**c**) plastic waste mix at 1000 °C/s heating rate, 1 bar, N_2_ atmosphere.

**Figure 6:**
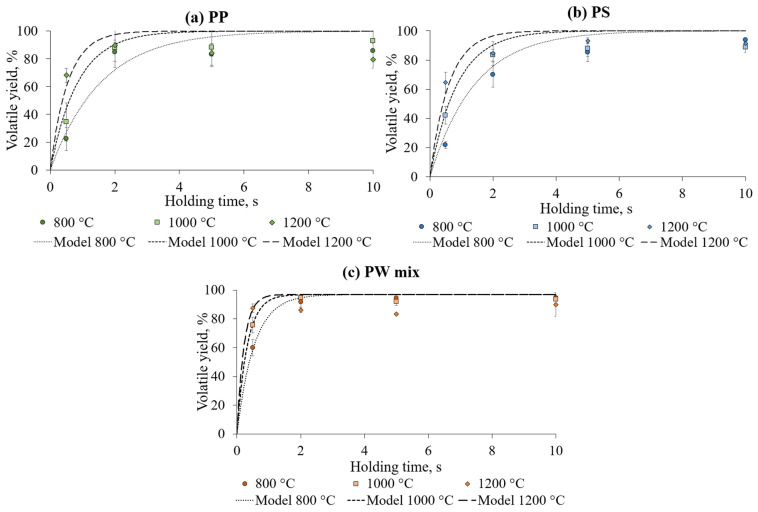
Devolatilisation models by SFOR, fitted to the measured data points.

Experimental volatile yields under isothermal pyrolysis conditions at 800–1200 °C and varied holding times are given in Figure 5. The volatile yield increases exponentially with pyrolysis temperature and logarithmically with holding time up to the maximal conversion value. Temperature elevation increases the reaction rate constant (as illustrated in Section 3.3, Figure 6), and the decomposition of polymeric chains is accelerated. The positive correlation between temperature and volatile yield holds up to 2 s for PP and PW mix samples and up to 5 s for PS samples (Figure 4). Within this time frame, the primary decomposition reactions dominate, and the system has not yet reached a plateau where the volatile release slows down due to the depletion of the substance. Systematic errors in the operation of WMR, introduced in the Section 4, determined the volatile yield levelling out at 90 ± 5%. The higher residual weight compared with that measured on the TGA (Figure 4) was also influenced to a lower extent by the deposition of pyrolytic carbon on the experimental meshes, as shown in Section 3.5.

Some previous kinetic studies focused on flash pyrolysis of plastics, although mostly in temperature ranges below 800 °C. PS (200 ± 20 µg, particle size of 200–500 μm) was reported to decompose in 15 s at 700 °C in a quartz tube Pyroprobe pyrolyser [53]. Kinetic data published on the flash decomposition of PP revealed that pyrolysis of PP (1 g, particle size of 1 mm) at 600 °C is completed in less than 70 s [57]. In fixed-bed systems, factors influencing the process (in addition to temperature), including particle size and sample weight, affect the heat distribution and, consequently, the reaction duration. In contrast, this study investigates the conditions corresponding to an entrained flow reactor, where the particles are heated from the reactor wall directly or hot carrier gas [58]; therefore, the determined devolatilisation times are considerably lower.

### 3.3. Kinetic Modelling of Devolatilisation

The data points from the WMR experiments (Figure 5) were used to develop models of the influence of temperature and residence time on the yields of primary cracking. A theoretical model was fitted to the empirical data for precise estimation of kinetic parameters. Devolatilisation profiles of studied plastics are presented in Figure 6.

The models illustrate the dependency of the primary cracking rate on temperature for different plastics, aiding in the estimation of conversion yield over the time frame. The activation energy for the process and the pre-exponential factor derived from the data in Figure 4 are presented in Table 3.

**Figure 7:**
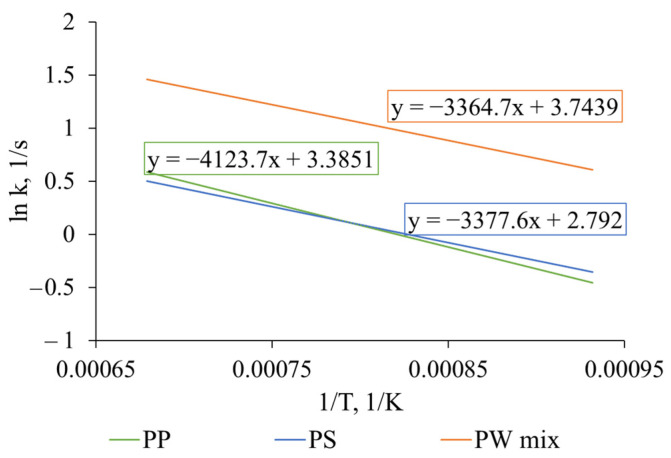
Arrhenius plot of PP, PS, and PW mix devolatilisation during flash pyrolysis—reaction rate dependence on temperature.

It was established that plastic packaging mix has a lower activation energy of decomposition and significantly higher reaction rate constants compared with pure plastics (Figure 7). The estimated activation energy of plastics pyrolysis decreased depending on the feed: PP > PS > PW mix. This decline in activation energy aligns with previous studies. Namely, A. F. Anene et al. [59] showed that PP and LDPE mix decomposes at 20 °C lower temperatures than pure LDPE. Additionally, the activation energy of pyrolysis of pure substances of higher thermal stability, PE, was reduced in the PP/PE/PS mix by 6%, as determined by T. Shan et al. [40]. This supports the hypothesis of physical interaction and a synergistic effect between polymers during co-pyrolysis, enhancing the decomposition process.

Figure 6 presents the Arrhenius plot of plastic primary cracking under flash heating conditions. It depicts the reaction rate constant’s dependence on the reaction temperature in a linear trend (when the logarithm of the k value is presented versus inverse temperature 1/T). Here, the activation energy can be determined by multiplying the slope of the line with the negative value of the gas constant (−R). Furthermore, the plot highlights the differences in the thermal behaviour of analysed feedstocks—the PP and PS decomposition rate is considerably slower than the PW mix decomposition rate at the same temperature.

As discussed in the introduction section, the estimates of kinetic parameters in non-isothermal pyrolysis using thermogravimetry in previous studies differ significantly from those calculated under flash pyrolysis conditions. The difference can be explained by the kinetic compensation effect, revealing the relationship between *E_a_* and *A* values, following the correlation (5) presented by the ICTAC Kinetics Committee [60]:

(5) $$ln\;A=aE_{a}+b$$

**Figure 8:**
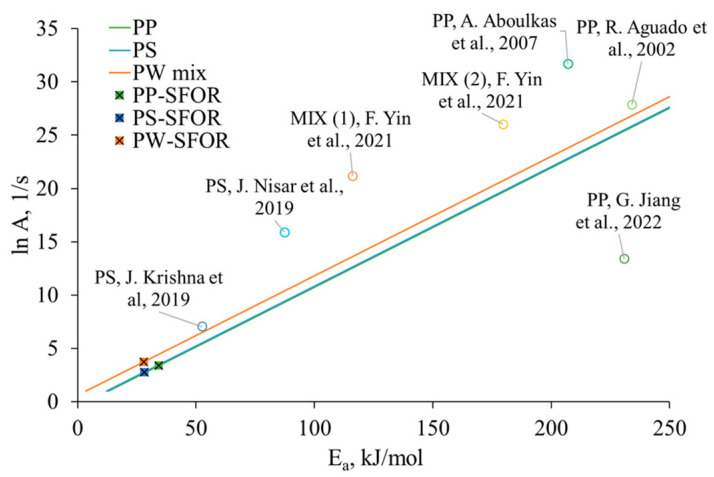
Kinetic compensation plot of plastic isothermal pyrolysis, where the linear functions correspond to the single value of k at 800 °C for the separate feedstocks [35,38,39,41,53,57].

Constants *a* (=1/RT_iso_) and *b* (=ln k_iso_) are calculated for the isokinetic temperature of the reaction, at which a series of ln *A* and *E_a_* have the same rate constant (k_iso_). To establish a linear relationship between kinetic parameters, *A* is expressed in logarithmic form. A higher *A* value, hence a larger ln *A*, suggests a higher rate of polymeric chain degradation initiation. The compensation plot in Figure 8 shows the *E_a_* of full devolatilisation reported for PS, PP, and PW mix in isothermal and non-isothermal pyrolysis correlated with the *A* factor reported in previous studies.

Only a limited number of kinetic studies have focused on isothermal high-temperature pyrolysis conditions for the decomposition of plastics. J.V. Krishna et al. [53] revealed that PS decomposition at 800 °C flash pyrolysis has a reaction rate constant of 0.21 s^−1^, with an estimated *E_a_* value of 52.6 kJ/mol. Data for flash pyrolysis of PP are available for the 400–600 °C temperature range, as reported by R. Aguado et al. [57]; at 600 °C, pyrolysis *k* = 0.013 s^−1^ and *E_a_* = 234 kJ/mol. On the other hand, numerous kinetic studies have been conducted on thermal analysis using low heating rates. Values of kinetic parameters defined by isoconversional approaches fall into a broad range, and the k values differ significantly from those estimated under isothermal conditions. Figure 8 indicates that the k values for plastic pyrolysis obtained in this study align closely with those reported in previous studies conducted under flash heating rates. Additionally, isoconversional values in the plots usually vary in activation energy as the pre-exponential factor is considered constant [61], which causes additional errors in kinetic parameter evaluation. The determination of *E_a_* and *A* for the set of data fitting in a range of temperatures ensured the precise estimation of the values in this study.

The reasons for the compensation effect for a specific reaction usually include experimental errors or incorrect model selection. Overall, regardless of whether the decomposition mechanism remains unchanged, the evaluation of kinetic parameters depends significantly on the experimental conditions selected [62]; therefore, in kinetic studies, selected process conditions, such as heating rate, temperature, atmosphere, and sample particle size, should closely match the target conditions in up-scale reactors where kinetic data will be applied.

### 3.4. Influence of Pressure on Volatile Yield During Flash Pyrolysis

**Figure 9:**
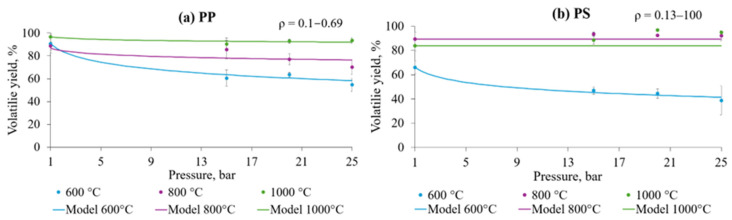
Devolatilisation of (**a**) PP and (**b**) PS at 600–1000 °C in 10 s under 1–25 bar pressure.

The influence of pyrolysis pressure on the volatile yield of pure plastics depended significantly on the set temperature in the 600–1000 °C range. Empirical data points and obtained devolatilisation models of plastic pressurised pyrolysis are given in Figure 9. PW mix was excluded from pressure dependence evaluation to avoid misinterpretation of the results, as the polymeric composition is unknown (considering a real municipal PW stream).

Pyrolysis pressure potentially had two effects on devolatilisation: decreased reaction rates and reduced volatile yield. The first suggests that samples maintained at pressurised conditions for sufficient time (>10 s) would reach the maximal conversion. The process was kinetically limited at 600 °C and 800 °C for PP and at 600 °C for PS, diminishing completely at 1000 °C for both feedstocks. Accordingly, estimated ρ values increased with temperature elevation, revealing a decrease in the impact of pressure. Reduced volatile yield at higher pressures during pyrolysis aligns with a theory of transport phenomena and vapour–liquid equilibrium effects. When total pressure increases, high molecular weight hydrocarbons (tars) fail to achieve adequate vapour pressure for evaporation and remain in the condensed phase [63]. However, as can be seen from the obtained results, above 800 °C, the average kinetic energy becomes sufficiently high for the removal of tars; therefore, increased pressure does not impact the VOC yield further. The volatile mass released from PS was affected by the pressure at 600 °C more considerably than PP due to the differing chemical structures. Polymers composed of aromatic monomers, such as polystyrene, tend to decompose and produce higher yields of tars, which stay in a condensed phase under pressure during thermal decomposition processes [64]; thus, full devolatilisation will not be reached in 10 s. The influence of pressure on the deposition of the CB was not established as the devolatilisation reached close to maximal value at ≥800 °C, with a 5–10% systematic error.

Previous studies on pressurised pyrolysis of plastics concentrated mainly at moderate temperatures of up to 600 °C. J. A. Onwudili et al. [65] found that PS is converted into liquid products, with a yield of 97 wt.% at 425 °C and 12.6 bar, and minor formation of char immediately; an increase of char to 3.4 wt.% in 120 min of reaction was reported, with pyrolytic oil remaining as the main product. The frequently highlighted optimal temperature for recovering ~97% of oil from PS is 425 °C [66]; however, when a target is a solid product, conversion requires sufficiently higher temperatures, reported at 900–950 °C [23]. A study of pressurised slow pyrolysis of the aliphatic polymer polyethene found that a pressure of 9.5–90 bar at 380 °C positively influenced the formation of aromatic hydrocarbons [67]. Although positively affecting the release of gases, no effect of pressure on the deposition of solid carbon was observed at this temperature.

### 3.5. Characterization of Pyrolytic Carbon Deposits by Raman Spectroscopy

**Figure 10:**
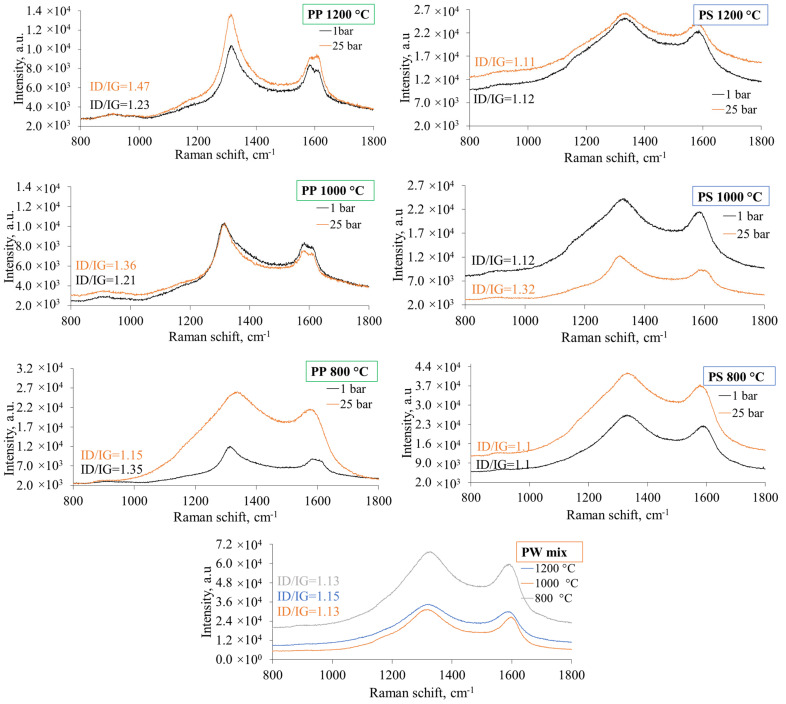
Raman spectra of pyrolytic carbon formed from PP and PS at 800–1200 °C, maintaining 1 bar and 25 bar pressures for 10 s; PW—carbon formed in non-pressurized pyrolysis.

Pyrolytic carbon particles and soft carbon filaments are deposited on the surface of wire meshes during plastic pyrolysis. Raman measurements were carried out to investigate the influence of pyrolysis temperature, pressure, and feedstock composition on the structural features of the formed carbon. The degree of graphitisation is one of the relevant parameters, indicating the degree to which carbon atoms are in hexagonal crystal configuration. In this study, it was assessed using Raman spectroscopy, with an inverse relationship to the degree of disorder (ID/IG value) estimating the defects within a graphitic structure; a higher ID/IG value indicates a greater structural disorder [68]. The first-order region of Raman spectra was recorded to estimate the intensity of the G band (Raman shift at 1580 cm^−1^), which is associated with a plane stretching motion in E2g modes of sp^2^-bonded carbon atoms of graphitic structures [69]. The other bands in this region correspond to point, or planar defects in the hexagonal lattice and thus are referred to as disorder bands, of which the most common are D1 and D2, which are present at around 1350 cm^−1^ and 1620 cm^−1^, respectively [70]. The disorder bands are typical for amorphous carbon, like furnace black. These characteristic bands were present in the Raman spectra of pyrolytic carbon, as illustrated in Figure 10.

Amorphous carbon with differing degrees of disorder formed during the flash pyrolysis of plastics. The relationship between feedstock composition and carbon’s structural characteristics was established. The formation of a less ordered CB structure was estimated for PP carbon compared to PS and PW feedstocks due to higher ID/IG values under almost all investigated conditions and a frequent presence of D2 peak. Raman measurement results in this study did not show a significant correlation between process temperature and pressure and disorder degree of carbon deposits. On the other hand, some carbon samples exhibited a reduced degree of graphitisation with increasing temperature. This effect was also established in the research on lignin-derived char. The disintegration of C=C bonds in char samples was determined at temperatures of 900 °C and above, with intense formation of amorphous carbon particles [71].

**Figure 11:**
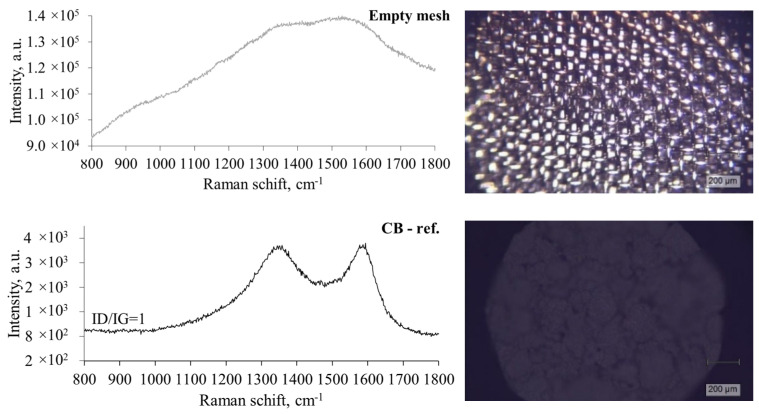
Raman spectra of an empty mesh and reference material, furnace black (CB-ref.). Images on the right were obtained with a Raman microscope objective lens of 5× magnification.

Raman spectra were measured for an empty mesh and a reference CB for comparison (Figure 10). The conventional carbon black (furnace black) exhibited similar structural characteristics to the pyrolytic carbon obtained in this study. The reference CB has relatively higher crystallinity than plastic-derived carbon, which has an ID/IG ratio of 1. Previous research on the structural characteristics of carbon black report that ID/IG values fall within the range of 0.91 to 1.4 [72,73]. The blank Raman measurement was the spectrum of an empty mesh (Figure 11) measured to accurately evaluate the origin of G, D1, and D2 peaks. The spectra of the empty mesh had a high-intensity fluorescence background. However, the characteristic bands for CB were not determined, confirming that the origin of the bands in post-experimental meshes was from pyrolytic carbon.

**Figure 12:**
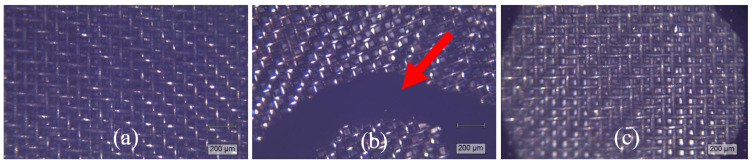
Pyrolytic carbon deposits at a magnification of 5×, derived from (**a**) PP, (**b**) PS, and (**c**) PW mix at 1000 °C and 1 bar.

The unavoidable influence of the catalytic effect of the mesh material (steel) determined the sedimentation of carbon on the meshes via chemical vapour deposition (Figure 12). This effect is supported by previous research by N. Gascoin et al. [74], who detected considerable deposition of coke from dodecane hydrocarbon pyrolysis on the surface of a stainless steel reactor at ~740 °C. Despite this, the special attribute of PS flash pyrolysis, which was not affected by the iron-based mesh surface, was the formation of soft filamentous carbon (Figure 12b, marked with a red arrow). Based on previous research, polystyrene decomposes through the rapid release of substantial yields of aromatic hydrocarbons [22,75,76], which is likely followed by polycondensation. The PS-derived carbon filaments displayed an ID/IG value of 1.12 (±0.05), in the range of that determined for CB.

## 4. Discussion

Possible sources of error in operating WMR arise primarily from difficulties distributing the sample in the mesh and the mass transfer of melted plastic to a cold zone (at the reactor electrodes), which causes an incomplete reaction. The temperature measurement has a standard deviation of 12.7 °C, and the error in setting the pressure is 0.25 bar. To ensure statistical significance, at least three repetitions were performed for each studied point.

The results of this study revealed that despite the chemical composition of the plastic feedstock, the primary cracking of plastic (weight of up to 30 mg) occurs in 2 to 5 s at 800–1200 °C considering entrained flow pyrolysis conditions. The secondary and tertiary reactions contributing to the deposition of CB at high temperatures and varied process pressures must be investigated further. It is already known that CB from toluene primarily deposits in 2 s at 950 °C in an inert ambient atmosphere [23]. The influence of the heterogeneous composition of primary hydrocarbons as feedstocks for CB production during pyrolysis must be considered as a factor influencing the particle properties, aggregate size in particular, determining the application field of the produced CB [11].

Collection of carbon black in a low-scale system, as employed in this research, was not feasible. L. F. Albright and J. C. Marek [23] conducted toluene high-temperature pyrolysis to obtain coke, highlighting the major deposition of carbon particles on the surface of the reactor at the exit end. Considering this pattern, the removal of the solid product should be performed by integrating quenching with water in this reactor zone for a scale-up [11].

L. F. Albright and J. C. Marek [23] emphasized the influence of pressure on toluene conversion—pressures below the atmospheric pressure (0.009–0.02 bar) at temperatures ≥900 °C favour coke formation. A hypothesis is raised that elevated pressure is a kinetically limiting factor for both primary cracking, as supported by the results of this study, and subsequent reactions resulting in CB deposition despite the promotion of aromatisation reactions. The influence of reduced pyrolysis pressure on the deposition of CB should be investigated further.

According to the results of the kinetic analysis and previously conducted research, the up-scale reactor configuration and process of the conversion of plastic into a CB product should consist of the following steps:

1. Cooled feeding (tubular) to feed plastics into the reaction temperature, avoiding melting (occurring at 100–200 °C) and clogging the feed inlet.
2. Entrained flow reactor tube of estimated length and flows targeting CB production with controlled CB aggregate sizes. The length of the reactor tube and the flow rate of N_2_ gas for primary decomposition and subsequent secondary and tertiary reactions—determining factors affecting reaction time.
3. Water quenching after pyrolysis to cool the reaction and collect the solid product.
4. Drying and characterisation of produced CB—the structural characteristics (graphitisation, crystallite size, and other) are measured by XRD or Raman spectroscopy; morphology of particles (size and morphology of single particles and aggregates)—measured by SEM or TEM microscopy methods. Determination of the composition of the solid product via ultimate and proximate analysis must be included.

## 5. Conclusions

This study investigated the devolatilisation of pure polypropylene and polystyrene plastics and plastic packaging waste as the first stage in their conversion to carbon black through high-temperature flash pyrolysis. The results revealed that the primary cracking is completed within 2 to 5 s at 800–1200 °C. Pyrolysis pressure was identified as a kinetically limiting factor of devolatilisation at lower temperatures, completely diminishing at ≥1000 °C. The deposited pyrolytic carbon had structural characteristics that closely resembled those of industrial carbon black, as confirmed by Raman spectroscopy.

Beyond the fundamental insights, this study has practical implications for sustainable carbon black production from plastic waste. The ability to produce high-quality carbon black from waste plastics presents opportunities for circular economy applications, reducing reliance on fossil-based feedstocks in industries such as tyre manufacturing, coatings, and inks. Furthermore, understanding the role of pyrolysis pressure and temperature provides a foundation for optimising reactor design and scaling up the process for industrial applications. Future research should focus on refining process parameters to enhance product quality, analysing secondary and tertiary reactions involved in carbon black formation from plastics, and evaluating the economic and environmental feasibility of large-scale implementation.

## Figures and Tables

**Table 1:** Proximate and ultimate analysis of plastic samples.

Parameter	PP	PS	PW Mix
Proximate analysis (wt.%)			
Moisture, %	–	–	–
Volatile matter, %	99.88	99.71	96.6
Fixed carbon *, %	0.12	0.29	2.2
Ash, %	–	–	1.2
Ultimate analysis (wt.%)			
C, %	86.28	94.94	77.3
H, %	13.72	5.06	12.47
N, %	–	–	0.11
S, %	–	–	0.05
O *, %	–	–	8.86
Cl, %	–	–	1.37

* By difference; – Below detection limit.

**Table 2:** Values of parameters used for pyrolysis model development.

Parameter	PP	PS	PW
*A* (1/s)	130	130	130
*E_a_* (J/mol)	48	48	48
*p_set_*, bar	1	1	1
*ρ*	100	100	100
α*	1	1	0.97
*α_pset_*	1	1	0.97

α*—material-specific maximal volatile matter share.

**Table 3:** Estimated values of Arrhenius parameters: *E_a_*—activation energy, *A*—pre-exponential factor during flash pyrolysis at atmospheric pressure.

	*E_a_*, kJ/mol	*A*, s^−1^
PP	34.3	29.52
PS	28.1	16.32
PW mix	28	42.26

## Data Availability

The original contributions presented in this study are included in the article. Further inquiries can be directed to the corresponding author.

## Abbreviations

PW mix	municipal plastic packaging waste
PP	polypropylene
PS	polystyrene
CB	carbon black
HC	hydrocarbons
VOC	volatile organic compound
WMR	wire mesh reactor
k	reaction rate constant
Ea	activation energy
A	pre-exponential factor
TGA	thermogravimetric analysis
DTG	differential thermogravimetric analysis
SFOR	single first-order reaction model
